# Prevention of Bone Metastases in Breast Cancer Patients. Therapeutic Perspectives

**DOI:** 10.3390/jcm3020521

**Published:** 2014-05-09

**Authors:** Philippe Beuzeboc, Suzy Scholl

**Affiliations:** Department of Medical Oncology, Institut Curie, 26 Rue d’Ulm, Paris 75005, France; E-Mail: suzy.scholl@curie.fr

**Keywords:** breast cancer, bone metastasis, adjuvant treatment, neoadjuvant treatment, bisphosphonates, zoledronic acid, denosumab, osteonecrosis of the jaw

## Abstract

One in four breast cancer patients is at risk of developing bone metastases in her life time. The early prevention of bone metastases is a crucial challenge. It has been suggested that the use of zoledronic acid (ZOL) in the adjuvant setting may reduce the persistence of disseminated tumor cells and thereby might improve outcome, specifically in a population of patients with a low estrogen microenvironment. More recently, the results of a large meta-analysis from 41 randomized trials comparing a bisphosphonate (BP) to placebo or to an open control have been presented at the 2013 San Antonio Breast Cancer Meeting. Data on 17,016 patients confirm that adjuvant BPs, irrespective of the type of treatment or the treatment schedule and formulation (oral or intra-venously (IV)), significantly reduced bone recurrences and improved breast cancer survival in postmenopausal women. No advantage was seen in premenopausal women. BPs are soon likely to become integrated into standard practice. Published data on the mechanisms involved in tumor cell seeding from the primary site, in homing to bone tissues and in the reactivation of dormant tumor cells will be reviewed; these might offer new ideas for innovative combination strategies.

## 1. Introduction

Metastatic breast cancer (MBC) remains an incurable disease. Around 70% to 80% of all MBC develop bone metastases. Their prevention represents therefore a major therapeutic goal. In spite of major advances in adjuvant therapies, combining hormonal, cytotoxic and anti-HER2 therapies for breast cancer over the last two decades [1,2,3,4,5], a substantial number of patients still experience relapse. A principal flaw may be that adjuvant treatments, directed against disseminated circulating tumor cells or micro metastatic emboli, are developed to target the increased proliferation rate of tumor cells as compared to normal cells, but thereby fail to eradicate dormant tumor cells. The dogma according to which only patients with HER2-amplified breast tumors would benefit from trastuzumab was challenged by a paper published by Paik and colleagues [6]. Controlled data from 172 cases included in the pivotal adjuvant National Surgical Adjuvant Breast and Bowel Project (NSABP)-B31 trial, whose tumors lacked *HER2* gene amplification, surprisingly were shown to benefit as much from adjuvant trastuzumab as did women whose tumors displayed HER2 amplification. Koraya *et al.* [7] have recently proposed that this potential clinical benefit of HER2 blockade in the adjuvant setting in tumors classified as HER2 might be explained by the hypothesis that the cancer stem cell expresses HER2.

The role of bone targeted therapies remains presently controversial in the adjuvant setting, while zoledronic acid (ZOL) or other bisphosphonates (BPs) and, more recently, denosumab (a RANK ligand monoclonal antibody) are widely used in the treatment of clinically detectable bone metastases, with the aim of directly blocking osteolysis [8]. ZOL, a third-generation BP characterized by an imidazole ring containing two nitrogen atoms, appears to be the most potent and widely used of the available nitrogen containing BPs (N-BPs). N-BPs inhibit osteoclast farnesyl pyrophosphate synthase activity, a key enzyme in the mevalonate pathway, which causes the inactivation of osteoclasts. 

This review focuses on the role of BPs and, more specifically, of ZOL, as well as of newer targeted treatments to prevent the settling of circulating micro metastases rather than to prevent the progression of established micro bone metastasis in patients with clinically “localized” breast cancer.

## 2. From Bench to Bedside

Tumor cell dissemination is parallel to tumor development [9], and tumor cells are easily detectable in blood and bone marrow aspirates (BMAs) at diagnosis. Patients with primary breast cancer frequently have evidence of minimal residual disease in the absence of clinical or radiological evidence of metastasis. The detection of circulating tumor cells (CTCs) in the blood and of disseminated cancer cells (DTCs) in the BMAs has now been well documented [10,11]. The patients with a high risk of relapse are not only more likely to have bone marrow DTCs at presentation [12], but also to demonstrate CTCs during follow-up [13]. However, while the detection of micro metastases in the bone marrow at the time of diagnosis of breast cancer is associated with a poor prognosis, their presence is not synonymous with inevitable relapse. Braun *et al.* [14] have analyzed individual patient data from nine studies involving 4703 Stage I–III breast cancer patients and evaluated the outcome over a 10-year follow-up period (median, 5.2 years). Micro metastases were detected in 30% of all patients. The presence of micro metastases was a significant prognostic factor with respect to poor overall survival (OS), breast-cancer-specific survival, disease-free survival (DFS) and distant-disease-free survival (DDFS) during the 10-year observation period. In a multivariate analysis, the presence of micro metastases was an independent predictor of a poor outcome. Persistence of DTCs in BMAs of breast cancer patients during follow-up visits predicts an increased risk for relapse. Pooled data from three trials involving 676 women with a primary diagnosis of breast cancer Stages I–III have been recently reported in a pan European study [15]. When BMAs were performed at a median of 37 months after the initial diagnosis and treatment of breast cancer, 15.5% of patients were found to have DTCs. The presence of DTC was an independent indicator of poor prognosis for DFS, cancer-specific survival and OS during the first five years following cancer diagnosis.

The time interval between initial treatment with curative intent and later clinical diagnosis of recurrent disease has been termed tumor dormancy. It is now commonly admitted that dormancy might be a steady state between slow growth and progressive apoptosis, with the result that tumor cells remain clinically undetectable [16]. After a variable period (in rare occasions, up to 20 years), cells can exit from dormancy, causing late relapse. The regulation of the switch from dormancy to clinical cancer progression in distant sites is poorly understood, but there is an extensive literature in the field [17]. Many factors have been invoked to contribute to persistence or interruption of cancer dormancy: Genetic and epigenetic changes, the angiogenic switch, micro environmental aspects, as well as immune surveillance cells [18].

The ability of a tumor to metastasize appears to be an inherent property of a subset of metastatic cancer stem cells (CSCs), as well as by changes induced by local micro environmental factors. Research in breast biology has provided support for a CSC hypothesis [19]. It has thus been postulated that tumors contain and are driven by a cell type that retains key stem-cell properties, including self-renewal. According to this model, subsequent cell differentiation steps occur that contribute to the clinically observed cellular heterogeneity. CSCs of breast tumor origin have been characterized by the following cell-surface phenotype cell differentiation markers: CD44+/CD24−. ALDH1 has more recently been described to be equally a marker of both normal and malignant human mammary stem cells and was shown to be a predictor of poor clinical outcome [20]. Balic *et al.* [21] have furthermore reported that most early CTCs detected in the bone marrow have a putative breast cancer stem cell phenotype. Niches for CSCs have been well characterized in the bone marrow [22], and the biologies of normal stem cells and CSCs appear to share remarkable similarities, which may have important implications when applied to the treatment of cancer metastasis [23]. Several lines of evidence argue in favor of CSCs to be responsible for therapy failure and disease recurrence. The corollary of this in the clinic is that if CSCs are the real culprits, their targeting is key for a definitive cure of cancer. Pathways that regulate breast cancer cells and, in particular, breast CSCs, such as Notch, Hedgehog and Wnt, provide new targets opportunities for therapeutic development [24,25,26,27]. In the EGFR pathway, *HER2* gene overexpression drives mammary carcinogenesis and invasion through its effects on mammary stem cells [28]. In a recent study, Ithimakin *et al.* [29] have shown that HER2 may equally play an important role in regulating the CSC population in luminal breast cancers where the primary tumor does not display HER2 amplification. It is assumed that HER2 is selectively expressed in and drives the CSC population. The RANK ligand is also able to regulate CSCs in luminal breast cancers through the induction of HER2 expression [30]. The recent demonstration that resistance to trastuzumab is mediated by an IL-6 dependent inflammatory loop has led to clinical trials with an anti-IL6 blocking reagent (tocilizumab) in an attempt to overcome *de novo* and acquired trastuzumab resistance [31]. Similarly, anti-HER2 treatment resistance can also be overcome by CXCR1 blockade using either a CXCR1-specific blocking antibody or repertaxin, a small-molecule CXCR1 inhibitor. Table 1 exposes ongoing clinical trials targeting CSCs.

**Table 1:** Cancer stem cell clinical trials.

Target	Agent/Class	Disease Site/Phase
Notch	Gamma secretase Inhibitor (GSI)	Breast/phase I + Taxotere
	Notch antibody	Phase I
	DLL-4 antibody	Phase I
Wnt	Frizzled antibody	Breast/phase I
Hedgehog	GSI + GDC-0449	Breast/phase I
HER2/Akt	Akt inhibitor + Lapatinib	Breast/phase I
CXCR1	Reparixin	Breast/phase I + Taxotere
IL6-R	Tocilizumab	Phase I

Targeting the homing, seeding and reactivation of CSCs in the potential metastatic niches at the initial time of tumor presentation could significantly inhibit disease progression. Blockade of the homing factor CXCR4 led an effective prevention of both primary tumor formation, as well as the resurgence of metastases in animal models [32]. Preventive actions against homing and seeding of CSCs might be technically difficult, since at the time of tumor diagnosis, CSCs may already have migrated to a pre-metastatic niche. Another potential clinical goal might be to block the reactivation of dormant CSCs at the metastatic sites. Future therapies based on this idea await a better characterization and validation of the dormancy model.

Secondary prevention of tumor progression by targeting tumor-associated macrophages (TAMs) might be planned [33]. Direct evidence of the role of TAMs in tumor progression is observed in breast cancer mouse models in which colony-stimulating factor-1 (CSF-1)-dependent TAM recruitment is required for tumor initiation through the stimulation of tumor angiogenesis [34,35]. At metastatic sites, inflammatory macrophages facilitate breast cancer cell extravasation, seeding and growth. Depletion of CSF-1 prevents TAM development. Early trials testing monoclonal anti-bodies directed against the CSF-1 or CSF-1 receptor are ongoing. Indirect evidence from so-called “stromal” gene signatures derived from human breast cancers are associated with a poor outcome. These stromal signatures are an independent prognostic marker and contain several highly expressed TAM-related genes [36]. Furthermore, CSF-1 is required for osteoclast maturation, while BPs and denosumab are potent inhibitors of osteoclast-mediated bone resorption and have demonstrated clinical utility in the treatment of patients with osteolytic bone metastases [37]. Both are known to reduce the risk of skeletal complications and prevent treatment-induced bone loss in patients with malignant bone disease. In early-stage breast cancer, adjuvant treatment with BPs has shown in some trials improvements in DFS and OS.

## 3. What Are the Experimental Data Concerning the Anti-Tumor Properties of Bisphosphonates and Especially Zoledronate?

Several experimental models have shown that the BPs can interfere with the growth of tumors and metastases in tissues other than the skeletal tissue [38]. The exact mechanisms responsible for the observed anti-tumor effects of BPs in preclinical models are unknown. Whether such effects *in vivo* are caused by a direct action on tumor cells or indirectly through inhibition of bone resorption remains unclear [39]. BPs can act directly on tumor cells by blocking tumor cell adhesion, invasion and proliferation and by inducing tumor cell apoptosis. Furthermore, BPs may also act indirectly on tumor cells through antiangiogenic [40] and/or immunomodulatory mechanisms [41].

Nevertheless, it is possible that the dominant anti-tumor activity of BPs in bone is caused via the inhibition of osteoclasts [42]. This could occur through the inhibition of bone resorption and consequent reduction in bone-derived growth factors that disrupt the inter-relationships between cancer, bone and hematopoietic stem cell populations, thereby creating a less favorable microenvironment for the survival of metastatic tumor cells [43].

BPs can act directly on tumor cells in combination with chemotherapy. Pre-clinical studies have demonstrated synergistic anti-tumor effects between chemotherapy agents commonly used in breast cancer treatment and N-BPs. In some animal models of breast cancer, ZOL showed a synergistic effect with some cytotoxic drugs, such as doxorubicin, and the sequence of dosing might be important [44,45].

## 4. What Are the Clinical Data Concerning the Anti-Tumor Properties of Bisphosphonates, Especially Zoledronate?

Provocative data have been first reported by Diel *et al.* [46] showing that adding oral clodronate to postoperative adjuvant breast cancer therapy could significantly improve DFS and OS in breast cancer patients with micro metastases to the bone marrow. Long-term follow-up data from their prospective, randomized, controlled study confirmed the initial results [47]. Patients with primary breast cancer received clodronate 1600 mg/day for two years or no treatment along with standard adjuvant breast cancer treatment. Analysis from 290 of 302 patients demonstrated that a significant improvement in OS was maintained in the clodronate group at a median follow-up of 103 +/− 12 months; 20.4% of patients in the clodronate group *versus* 40.7% of control group patients (*p* = 0.04) died during the 8.5 years following primary surgical therapy.

Nevertheless, a subsequent meta-analysis [48] did not detect evidence of any statistically significant difference in OS, bone metastasis-free survival or non-skeletal metastasis-free survival in advanced breast cancer patients receiving clodronate therapy or early breast cancer patients receiving adjuvant clodronate treatment compared with those who did not receive any active treatment. These inconclusive results from adjuvant trials of the oral clodronate, in the 1990s, formed the rationale for adjuvant trials of the more potent ZOL. It was anticipated that ZOL might have more definite beneficial anti-tumor effects.

Lessons learned from a neoadjuvant treatment with ZOL, especially from the NEO-Adjuvant Zoledronic Acid to Reduce Recurrence (AZURE) trial showed a potentially synergistic effect in combination with chemotherapy. Winter *et al.* [49] have explored the potential synergistic anti-tumor effects of the sequential treatment of neoadjuvant chemotherapy followed by ZOL in a pilot randomized Phase II study. Biological endpoints included apoptosis (apoptotic index), proliferation (KI67) and angiogenesis (serum VEGF) in patients with breast cancer. Short-term changes in biomarkers suggest possible complex interactions between both treatments, but need to be confirmed.

In the retrospective NEO-AZURE study [50], the combination of chemotherapy followed by ZOL compared to chemotherapy alone significantly reduced the residual invasive tumor size (RITS) at surgery (*p* = 0.006). However, there was no significant difference in pathological complete response (pCR), 11.7% *vs*. 6.9%, respectively (*p* = 0.146). Unfortunately, in the clinical neoadjuvant setting, there are no other published data regarding the potential anti-tumor effect of ZOL in combination with chemotherpay (CT). Nevertheless, these intriguing results with a limited population of patients warrant formal evaluation in large prospective studies.

In the first randomized post-neoadjuvant NATAN study, treatment with ZOL compared to observation in 693 patients without a pathologic complete response did not improve outcome after anthracycline-taxane-based chemotherapy for primary breast cancer. A non-significant trend favoring ZOL in patients >55 years was found [51].

Some results of Phase II randomized studies have indicated a potential antineoplastic effect of ZOL on persisting DTCs. In the controlled study reported by Solomayer *et al.* [52], 96 patients with early breast cancer (BC) and DTC-positive bone marrow were randomized to treatment with ZOL plus adjuvant systemic therapy or adjuvant systemic therapy alone. DTC-positive patients treated with ZOL were more likely to become DTC-negative after 12 months of treatment compared with the controls (67% *versus* 35%; *p* = 0.009). Aft *et al.* [53] have shown that ZOL administered with chemotherapy resulted in a decreased proportion of patients with DTCs detected in the bone marrow at the time of surgery three months later. In a matched-pair study reported by Rack *et al.* [54], while DTCs were detected in 172 patients at the time of first BMA, DTCs were detected in only 13% of the 31 patients treated with ZOL in contrast with 27% of the 141 patients in the control group (*p* = 0.099). This improvement of elimination of DTCs due to treatment with ZOL need to be confirmed.

The Austrian Breast and Colorectal Cancer Study Group (ABCSG)-12 trial and the Zometa-Femara Adjuvant Synergy Trial (ZO-FAST) were in concordance regarding the addition of ZOL to adjuvant endocrine therapy, both showing a significant benefit in DFS. In the ABSCG-12 trial, 1803 premenopausal women with endocrine-receptor-positive early-stage (Stage I–II) breast cancer receiving goserelin were randomized to receive anastrozole or tamoxifen with or without ZOL (4 mg every six months) for three years. Analysis at the 48 months’ follow-up [55] showed that the addition of ZOL to adjuvant endocrine therapy significantly improved DFS in bone and other sites. At a median follow-up of 62 months [56], ZOL reduced the risk of DFS events by 32% (HR = 0.68, 95% CI 0.51–0.91; *p* = 0.009).

In the final 60-month results of the ZO-FAST study [57], immediate ZOL reduced the risk of DFS events by 34% (HR = 0.66; *p* = 0.0375) with fewer local (0.9% *versus* 2.3%) and distant (5.5% *versus* 7.7%) recurrences *versus* delayed ZOL.

Unfortunately, the findings of the large AZURE trial [58] do not confirm any improvement in DFS or OS in the analysis of the whole population of the study and do not support the routine use of ZOL in the adjuvant management of breast cancer. In this open-label Phase III study, 3360 postmenopausal and non-postmenopausal breast cancer patients were randomly assigned to receive standard adjuvant systemic therapy either with or without ZOL. ZOL was administered every three to four weeks for six doses and, then, every three to six months to complete five years of treatment. At a median follow-up of 59 months, there was no significant difference in the primary end point, with a rate of DFS of 77% in each group. The numbers of deaths were also similar, resulting in rates of OS of 85.4% in the ZOL group and 83.1% in the control group.

To address whether the use of ZOL in the adjuvant setting of breast cancer might have any effect on the natural course of the disease and which group of patients might benefit, Yan *et al.* [59] have performed a meta-analysis of five randomized controlled trials (Table 2). Among these trials, the patients of Z-FAST, ZO-FAST and E-ZO-FAST trials [60,61,62] were postmenopausal women, whereas the ABCSG-12 trial included premenopausal women treated with ovarian suppression by goserelin. The AZURE trial enrolled both postmenopausal and non-postmenopausal women.

**Table 2:** Characteristics of the main five adjuvant randomized studies with zoledronic acid. ZOL, zoledronic acid.

Author (trial)	Patients per arm	Regimen	Dosage ZOL	Combination therapy	Duration (years)	Median FU (months)
Brufsky [51]	300	Immediate ZOL	4 mg IV/6 months	Letrozole	5	60
(Z-FAST)	300	Delayed ZOL				
Coleman [48]	532	Immediate ZOL	4 mg IV/6 months	Letrozole	5	60
(ZO-FAST)	533	Delayed ZOL				
Llombarto [53]	263	Immediate ZOL	4 mg IV/6 months	Letrozole	5	36
(E-ZO-FAST)	264	Delayed ZOL				
Gnant [47]	900	ZOL	4 mg IV/6 months	Goserelin +	3	62
(ABCSG 12)	903	No treatment		Tamoxifen or Anastrozole		
Coleman [49]	1861	ZOL	4 mg IV/3–4 weeks ×6	Standard	5	59
(AZURE)	1678	No treatment	then 3 monthly ×8 and 6 monthly ×5	adjuvant treatment		

This meta-analysis indicated that ZOL did not significantly improve the prognosis of breast cancer patients. However, a subgroup analysis suggested that adjuvant use of ZOL could significantly improve DFS (pooled RR = 0.763, 95% CI, 0.658–0.884; *p* < 0.001) and reduced the risk of locoregional and distant recurrence in postmenopausal breast cancer patients. These conclusions are concordant with those from the pre-stratified subgroups analysis of the AZURE study. In this trial, for postmenopausal (menopause starting more than five years earlier) patients, the rates of invasive-DFS at five years were 78.2% in the ZOL group and 71.0% in the control group (HR = 0.75; 95% CI, 0.59 to 0.96; *p* = 0.02) and the five-year OS rates were, respectively, 84.6% and 78.7% (HR = 0.74; 95% CI, 0.55 to 0.98; *p* = 0.04). This meta-analysis has many limitations. First, all of the trials focused on ER-/PR-positive patients treated with different adjuvant endocrine therapy with the exception of AZURE trial. Secondly, some of the patients in the control group were those treated with delayed ZOL, and therefore, the effect of ZOL might be underestimated in the treatment group.

A meta-analysis of individual data from 17,016 patients included in 41 randomized trials that compared BP to no BP (placebo or open control) has been very recently presented by R. Coleman at the 2013 San Antonio Breast Cancer Symposium [63]. It confirms that adjuvant BPs significantly reduce bone recurrences (HR = 0.66, 10-year gain = 2.9%) and improve breast cancer survival (HR = 0.83, 10-year gain = 3.1%) in postmenopausal, but not in premenopausal, women. Reductions in bone recurrence for postmenopausal women were similar, irrespective of BP type, treatment schedule, ER status, nodal involvement or the use of concomitant chemotherapy. There were no improvements in bone (RR = 1.00) or other recurrence for premenopausal women.

The risk of osteonecrosis of the jaw (ONJ) with BPs in the adjuvant setting is very low. Mauri *et al.* [64] have published the results of a meta-analysis of 15 studies reporting data on ONJ. ONJ was a rare event, reported in only 13 (0.24%) of the 5312 patients receiving BPs, and in one of the 5382 patients in the control group. All of the 13 events of ONJ occurred in patients undergoing treatment with ZOL. In the AZURE trial, there were 17 confirmed cases of ONJ (cumulative incidence: 1.1%).

## 5. What Is the Potential Role of Denosumab?

RANKL and RANK play a critical role in the expansion of tumor cells in bone. Preclinical studies demonstrated that inhibition of RANKL significantly delays skeletal tumor formation, reduces skeletal tumor burden and prolongs the survival of tumor-bearing mice [65].

In the metastatic setting, data collected and combined from three identically designed Phase III trials of patients with breast cancer [66], prostate cancer, other solid tumors or multiple myeloma have shown that denosumab was superior to ZOL in reducing the risk of a first skeletal-related event with a median delay of 8.3 months [67]. The efficacy of denosumab was maintained for multiple events and in patient subpopulations. However, disease progression and OS were similar between the both treatments.

In the adjuvant setting, denosumab and ZOL have never been compared, but two large, randomized, double-blind, placebo-controlled Phase III trials are ongoing to evaluate the potential therapeutic role of denosumab in patients with non-metastatic breast cancer receiving aromatase inhibitor therapy (Austrian Breast and Colorectal Cancer Study Group 18 (ABCSG-18), *n* = 3400) and in women with early stage cancer at high risk of recurrence (D-CARE, *n* = 4500). The D-CARE trial is designed to assess if denosumab treatment prolongs bone metastasis-free survival. Secondary endpoints include disease-free survival (DFS) and overall survival. High risk is defined as biopsy evidence of breast cancer in regional lymph nodes, tumor size >5 cm (T3) or locally advanced disease (T4). Patients are randomized to receive denosumab 120 mg or placebo subcutaneously monthly for six months, then every three months for a total of five years of treatment. Both ABCSG-18 and D-CARE trials have finished accrual.

In men with castrate-resistant prostate cancer, denosumab compared to placebo significantly prolonged bone metastasis-free survival and delayed time to first bone metastasis in a large Phase III study [68].

Importantly, denosumab induced a striking tumor response rate in patients with giant cell tumors of bone. In these tumors, neoplastic stromal cells express high concentrations of RANKL and activate RANK-positive osteoclast-like giant cells. Chawla *et al.* [69] have recently reported the safety and efficacy of denosumab in more than 200 patients.

Considering the potential role of RANKL on RANK-expressing breast cancer cells in preclinical models, denosumab could exhibit antitumor effects in patients. Furthermore, RANK expression in primary tumors might be a predictive marker of bone metastasis. In an independent series of 93 primary tumors [70], immunohistochemical analysis of RANK showed a positive correlation with the development of bone metastases and a shorter skeletal DFS. This approach might help to select patients for adjuvant treatment.

## 6. What Are the Potential Roles of Integrin and Src Inhibitors [71]?

It is now well accepted that αvβ3 integrin is a central molecule for osteoclast function. Therapeutics targeting αvβ3 would be particularly promising for the treatment of advanced cancers associated with skeletal lesions, because these drugs could inhibit bone metastasis formation [72]. Antibodies targeting αvβ3 integrin are under way to treat cancers associated with skeletal lesions.

The Src pathway is active in breast cancer and promotes cell proliferation, invasiveness and metastases [73,74]. Dasatinib inhibits the protein tyrosine kinase, Src, which can support the development of bone metastases in patients with ER+ breast cancer.

Preclinical data support the use of dasatinib to inhibit breast cancer cell growth; however, early Phase II studies to date have shown only limited responses to dasatinib and bosutinib alone in MBC patients [75,76,77]. The addition of dasatinib to letrozole in MBC patients receiving their first aromatase inhibitor therapy for metastatic disease did not improve the clinical benefit rate compared with letrozole alone in a Phase II randomized trial, but median PFS improved from 11 to 22 months (*p* = 0.05) with the addition of dasatinib, suggesting dasatinib improved the duration of disease control combined with letrozole [78]. These preliminary results need to be confirmed in a large Phase III trial.

The role of dasatinib in combination with paclitaxel to prevent the progression of disease in bone in MBC patients is currently being explored for the treatment of metastatic breast cancer at the Memorial Sloan Kettering Cancer Center, New York, NY, USA.

In metastatic castrate-resistant prostate cancer patients, the combination of dasatinib to docetaxel compared to docetaxel alone failed to increase OS in the large Phase III STEADY trial [79].

## 7. Conclusions and Perspectives

Many data strongly support the use of adjuvant bone-targeted treatments [80]. The effect of BPs in breast cancer adjuvant therapy concerning the improvement of patient survival limited to patients with a low estrogen microenvironment is now confirmed in a large meta-analysis. However, the underlying mechanism is unclear and further research is needed.

A better definition and understanding of the prognostic factors should allow the identification of patients at high risk of relapse to develop new strategies in order to improve prognosis. Using gene microarray and bioinformatics analysis, Smid *et al.* [81] found that tumors with a luminal subtype gene signature were more likely to develop bone metastasis. Besides CTCs and DTCs, circulating free DNA (cfDNA) should be used in future trials for the monitoring of new adjuvant or neoadjuvant therapies [82]. Advances in sequencing technologies have enabled the rapid identification of somatic genomic alterations in individual tumors. cfDNA, believed to be derived from tumor cell necrosis and lysis, sharing similar genetic features to primary tumor, can be detected in the blood of breast cancer patients. Some studies have already shown the feasibility of using cfDNA to monitor tumor dynamics in metastatic patients [83].
